# Radiotherapy Side Effects: Comprehensive Proteomic Study Unraveled Neural Stem Cell Degenerative Differentiation upon Ionizing Radiation

**DOI:** 10.3390/biom12121759

**Published:** 2022-11-26

**Authors:** Dong Liang, Meng Ning, Hang Xie, Xiaoyan He, Peigen Ren, Xiaohua Lei, Xuepei Zhang

**Affiliations:** 1 Center for Energy Metabolism and Reproduction, Institute of Biomedicine and Biotechnology, Shenzhen Institutes of Advanced Technology, Chinese Academy of Sciences, Shenzhen 518055, China; 2 Department of Endocrinology, First Affiliated Hospital of Harbin Medical University, Harbin 150001, China; 3 Department of Structural Heart Disease, The First Affiliated Hospital of Xi’an Jiaotong University, Xi’an 710061, China; 4 Shanghai Key Laboratory of Molecular Imaging, School of Pharmacy, Shanghai University of Medicine & Health Sciences, Shanghai 201318, China; 5 State Key Laboratory of Bioorganic and Natural Products Chemistry, Center for Excellence in Molecular Synthesis, Shanghai Institute of Organic Chemistry, Chinese Academy of Sciences, Shanghai 200032, China

**Keywords:** radiation side effects, neural stem cell, redox proteomics

## Abstract

Cranial radiation therapy is one of the most effective treatments for childhood brain cancers. Despite the ameliorated survival rate of juvenile patients, radiation exposure-induced brain neurogenic region injury could markedly impair patients’ cognitive functions and even their quality of life. Determining the mechanism underlying neural stem cells (NSCs) response to irradiation stress is a crucial therapeutic strategy for cognitive impairment. The present study demonstrated that X-ray irradiation arrested NSCs’ cell cycle and impacted cell differentiation. To further characterize irradiation-induced molecular alterations in NSCs, two-dimensional high-resolution mass spectrometry-based quantitative proteomics analyses were conducted to explore the mechanism underlying ionizing radiation’s influence on stem cell differentiation. We observed that ionizing radiation suppressed intracellular protein transport, neuron projection development, etc., particularly in differentiated cells. Redox proteomics was performed for the quantification of cysteine thiol modifications in order to profile the oxidation-reduction status of proteins in stem cells that underwent ionizing radiation treatment. Via conjoint screening of protein expression abundance and redox status datasets, several significantly expressed and oxidized proteins were identified in differentiating NSCs subjected to X-ray irradiation. Among these proteins, succinate dehydrogenase [ubiquinone] flavoprotein subunit, mitochondrial (sdha) and the acyl carrier protein, mitochondrial (Ndufab1) were highly related to neurodegenerative diseases such as Parkinson’s disease, Alzheimer’s disease, and Huntington’s disease, illustrating the dual-character of NSCs in cell differentiation: following exposure to ionizing radiation, the normal differentiation of NSCs was compromised, and the upregulated oxidized proteins implied a degenerative differentiation trajectory. These findings could be integrated into research on neurodegenerative diseases and future preventive strategies.

## 1. Introduction

Radiation therapy (RT) is one of the most effective treatments for primary and secondary brain tumors in adult and pediatric patients. However, cranial irradiation induces cognitive decline and intellectual dysfunction, such as impaired learning and memory. The adverse effects are more pronounced in children, especially when the temporal lobe, where the hippocampus is located, is irradiated [1,2,3,4,5]. Due to the widespread application of RT treatment, the quality of life of an expanding number of long-term survivors is garnering increasing concern.

Neural stem cells (NSCs) in the hippocampus are capable of self-renewal and differentiation into neurons, astrocytes, and oligodendrocytes [6,7]. Contrary to mature neurons, which are considered to be in an irreversible state of growth arrest, the rapidly dividing and undifferentiated NSCs are more susceptible to irradiation. Several studies have indicated that irradiation of the hippocampus induced apoptosis in the subgranular zone of the Dentate gyrus (DG) [8], diminished the proliferation of the surviving NSCs [9], and impeded the differentiation of NSCs into neurons [10]. These irradiation-induced alterations which inhibit neurogenesis have been implicated in cognitive impairment [11,12,13], and elucidating the mechanisms underlying damage to NSCs could enable the discovery of strategies to optimize cognitive brain function and lessen RT-induced adverse effects.

Similarly, to various other cellular stress factors, ionizing radiation damages DNA strands by disrupting their sugar-phosphate backbone and induces overall cellular toxicity, thereby driving cells towards apoptosis, necrosis, autophagy, or senescence [14,15,16,17,18,19,20,21,22,23]. Another consequential effect of irradiation on cellular macromolecules is the generation of reactive oxygen species (ROS) and reactive nitrogen species (RNS), which are predominant sources of damage to normal tissue [24,25]. There are reports that the reduction and oxidation (redox) systems play a critical role in acute radiation syndrome and are responsible for several early and late-stage side effects [26,27]. To date, published studies have elucidated the influence and functions of free radicals in radiation-induced pressure, as well as the association between redox and mitochondrial functions. Notably, some studies applied the redox theory to discover new chemicals to enhance RT sensitivity [28,29,30,31]. However, due to the characteristics of protein modification, it is challenging for conventional omics research to profile the transcriptomic and proteomic variations in cells undergoing a redox process. The redox states of whole cellular proteins in irradiated NSCs still remain unclear.

In the present study, mouse neural stem cells were exposed to X-ray irradiation to establish the cell stress model; concurrently, fetal bovine serum (FBS) was utilized to induce differentiation. iodoTMT was employed to label-free sulfhydryl groups on cysteine residues; a proteome-wide screening was conducted, followed by a comprehensive analysis of the redox patterns. Differentially expressed proteins were identified in NSCs subjected to X-ray irradiation and induced to differentiate. From a redox-MS perspective, under sustained irradiation-induced pressure, NSCs’ natural differentiation capability could be disrupted. Furthermore, the emergence of heavily oxidized proteins in NSCs was indicative of these cells’ susceptibility to degeneration.

## 2. Results

### 2.1. NSC Proteomic Pattern Profiling Following Different Treatments

**Figure 1:**
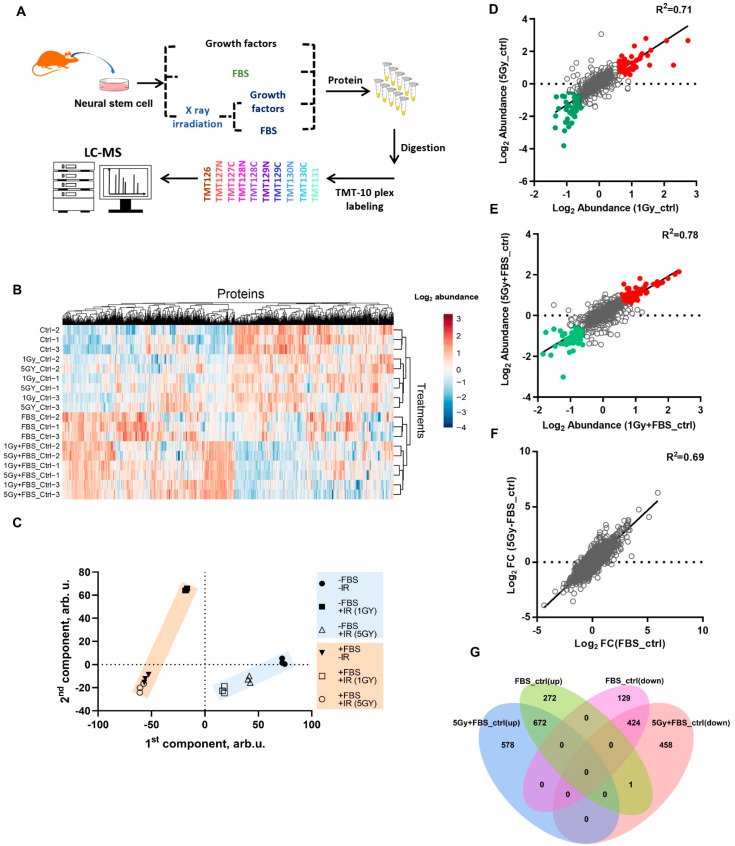
Overview of the experimental design and protein expression profiles. (**A**) Schematic depiction of experimental grouping and proteomic analysis workflow. (**B**) Heat maps of the differentially expressed proteins in each group, the proteomic detections were performed 36 h after each treatment (red, upregulated; blue, downregulated). (**C**) Principal component analysis (PCA) of proteomic expression profile among groups; six groups were separated by two main factors: the presence or absence of FBS. “–FBS–IR” represents the “ctrl” group, “–FBS+IR” denotes the “1Gy_ctrl or 5Gy_ctrl” group, “+FBS–IR” represents the “FBS_ctrl” group, and “+FBS+IR” represents the “1Gy+FBS_ctrl or 5Gy+FBS_ctrl” group. (**D**) Scatter plot of protein abundance correlation between the 1Gy_ctrl and 5Gy_ctrl groups. (**E**) Scatter plot of protein abundance correlation between the 1Gy+FBS_ctrl and 5Gy+FBS_ctrl groups. (**F**) Scatter plot of protein abundance correlation between the FBS_ctrl and 5Gy+FBS_ctrl groups. (**G**) Venn diagram illustrating the overlap of up or downregulated proteins among different groups.

In order to elucidate the proteomic influence of irradiation on neural stem cell (NSC) proliferation and differentiation, the present study is designed in the following way (Figure 1A). The embryonic mouse brain derived NSCs were initially maintained in the medium supplemented with a cocktail of growth factors. Subsequently, the NSCs were divided into four treatment-specific groups: NSCs cultured with growth factors without additional treatment (“ctrl”); growth factor cocktail medium replaced by DMEM+FBS for differentiation (“FBS_ctrl”); NSCs cultured with growth factors subjected to a dose gradient X-ray irradiation, but without differentiation induction (“1Gy_ctrl” and “5Gy_ctrl”); and growth factors replaced with DMEM+FBS immediately following irradiation (“1Gy+FBS_ctrl” and “5Gy+FBS_ctrl”). The “1Gy+FBS_ctrl” and “5Gy+FBS_ctrl” group’s purpose was to investigate the impact of irradiation on NSCs’ differentiation. Approximately 6300 proteins were identified by mass spectrometry in the different groups, and a heatmap of the protein expression profile indicated that the treatments induced proteomic alterations in NSCs (Figure 1B). Principal component analysis (PCA) of the entire proteome dataset revealed that FBS and irradiation affect protein expression patterns. Two distinct clusters were distinguishable based on FBS treatment, and within each group, X-ray irradiation caused a further division into subgroups (Figure 1C); these indicated that FBS was the principal influencing factor. Afterward, a correlation analysis based on group-specific protein expression data was performed. Significant correlations were observed between 1 Gy and 5 Gy treatments for different radiation doses with or without FBS stimulation (Figure 1D,E).

Notably, significant correlations also existed between FBS with IR and FBS without IR (Figure 1F). Consistent with PCA, these indicated that FBS treatment, which promotes cell differentiation, was the predominant contributor to the protein expression pattern in NSCs, and that irradiation did not impact the proteomic profile. In order to clarify the role irradiation might play in NSCs’ proliferation and differentiation, further proteome data mining was conducted. Venn analysis illustrated that compared with the control group, in both the post-IR differentiation (5Gy+FBS_ctrl) and untreated differentiation groups (FBS_ctrl), 1096 proteins were similarly altered, of which 672 were upregulated and 424 were downregulated. In the upregulated proteins fraction, a considerable number of differentially expressed proteins (DEPs) were observed: 578 proteins were exclusive to the 5Gy+FBS_ctrl group, and 272 proteins belonged to the FBS_ctrl group. The downregulated protein fraction demonstrated a comparable phenomenon (Figure 1G). Information on these DEPs would facilitate the quest to unravel the protein interaction networks of IR-induced effects on NSCs differentiation.

### 2.2. Functional Annotation of Differentially Expressed Proteins

**Figure 2:**
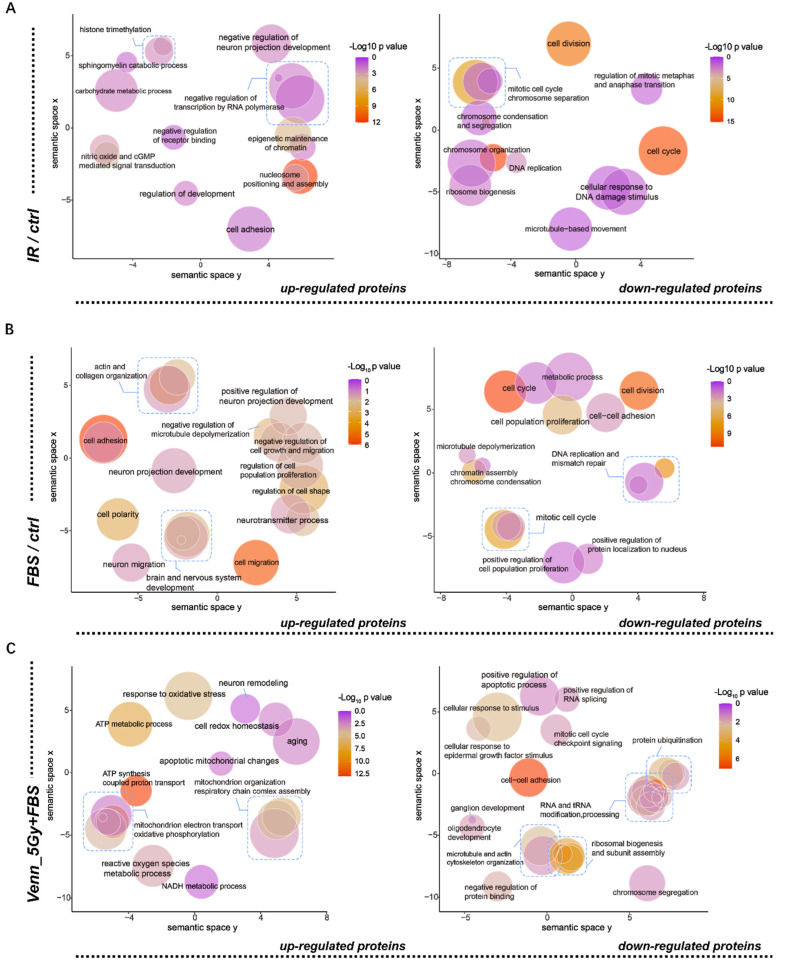
Gene ontology (GO) enrichment analysis and visualization of differentially expressed proteins. (**A**–**C**) The scatter plots show the significantly enriched GO terms for DEPs in different groups, (**A**) IR vs. ctrl group, (**B**) FBS vs. ctrl group, and (**C**) 5Gy+FBS vs. FBS group.

In order to determine the biological functions of the differentially expressed proteins screened above (Figure 1G), gene ontology (GO) analysis was performed. Following treatment with X-ray only, the upregulated proteins in NSCs were implicated in cell adhesion, negative regulation of neuron projection development and nitric oxide, etc.; conversely, the downregulated proteins were enriched in cell division, the cell cycle, and DNA replication (Figure 2A). These are indicative of the overall adverse effects of irradiation on cells.

When NSCs were treated with FBS, the upregulated proteins were predominantly enriched in neuron projection development, cell polarity, and negative regulation of cell growth and migration; meanwhile, the downregulated proteins were associated with the cell cycle, cell division, and cell proliferation, which was representative of the pro-differentiation effects of FBS (Figure 2B). Subsequent analysis focused on comparisons of DEPs between the FBS individual treatment group and the post-IR FBS treatment group. As described in Figure 2C, the upregulated proteins in the 5Gy+FBS group were principally enriched in oxidative stress, aging, mitochondrial alterations, and neuron remodeling. The downregulated proteins were specific for RNA processing, cell development, cell adhesion, and the mitotic cell cycle. The above findings demonstrated that irradiation impacted NSC proliferation and differentiation, and the most probable mechanism underlying this influence is oxidative stress. The redox patterns of post-IR differentiation were further investigated in the next analysis.

### 2.3. Irradiation Influenced the Proliferation Capacity, Cell Cycle, and Stemness of NSCs

**Figure 3:**
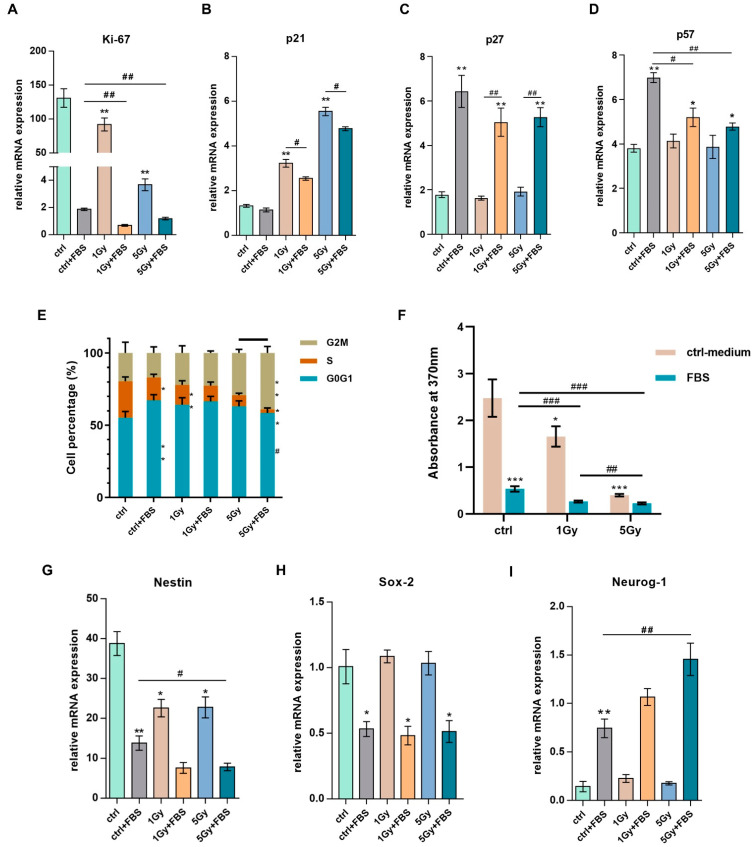
IR impacted NSC proliferation and their cell cycle. (**A**–**D**) Representations of the relative mRNA expression of proliferation markers of NSCs under different treatments. (**E**) A histogram represents the percentage of NSCs in the different phases of the cell cycle for the indicated treatments. (**F**) A bar graph with absorbance value representing the amount of BrdU incorporated in newly synthesized cellular DNA. (**G**–**I**) Bar graph showing the relative mRNA expressions of neural progenitor markers of NSCs undergoing different treatments for 24 h. *n* = 3; Mean ± SEM. * *p* < 0.05, ** *p* < 0.01, *** *p* < 0.001 as compared with the control; # *p* < 0.05, ## *p* < 0.01, ### *p* < 0.001 for comparisons between indicated groups.

For a comprehensive determination of the effects of IR on NSCs’ properties, cell cycle and proliferation assays were conducted, and the expression levels of the relevant genes were analyzed. Irradiation and FBS treatment inhibited NSC proliferation and decreased Ki-67 expression; the suppressive effects were more pronounced in the 5Gy+FBS group (Figure 3A). A similar phenomenon was also observed in the BrdU proliferation assay (Figure 3F). p21, which engenders cell arrest following DNA damage, exhibited a dose-dependent upregulation 24 h after X-ray irradiation, and FBS treatment diminished the increase in p21 (Figure 3B). The mRNA expressions of two other cyclin-dependent kinase inhibitors (CKIs), p27 and p57, were unaffected by X-ray but upregulated by FBS. Since increasing p27 and p57 have been reported to be associated with cell differentiation [32,33], the downward trends in the IR + FBS groups indicated the impact of X-ray intervention on cell differentiation (Figure 3C,D); no significance was observed for p27, but p57 decreased significantly in IR + FBS group in comparison with ctrl + FBS group. Furthermore, the cell cycle phases of NSCs were also affected by X-ray and FBS. Both irradiation and FBS hindered DNA synthesis, thereby occasioning S phase-inducing arrest (Figure 3E), while the FBS group presented with a relatively longer G1 phase which was indicative of continuous cell development [34]. Combination treatment with irradiation and FBS interventions demonstrated stronger suppression at the S phase and a reduced population at G1 (Figure 3E), implying that irradiation disrupted the normal differentiating cell cycle patterns of NSCs. FBS promoted NSC differentiation and affected cell pluripotency, as evidenced by the downregulation of Nestin and Sox2 and upregulated Neurog-1 (Figure 3G–I). Compared to the FBS group, the irradiation group revealed moderate impacts on neural progenitor identity-related genes. When NSCs were subjected to X-ray, nestin was downregulated, while Sox2 and Neurog-1 remained unchanged (Figure 3G–I). In summary, both irradiation and differentiation influenced NSCs’ cell cycle and the expression of stem cell marker genes, albeit differently. The X-ray-induced aberrant cell cycle reflects the detrimental impact of irradiation on NSC differentiation.

### 2.4. Irradiation Impeded NSC Differentiation and Altered Neurogenesis-Associated Protein Expression

**Figure 4:**
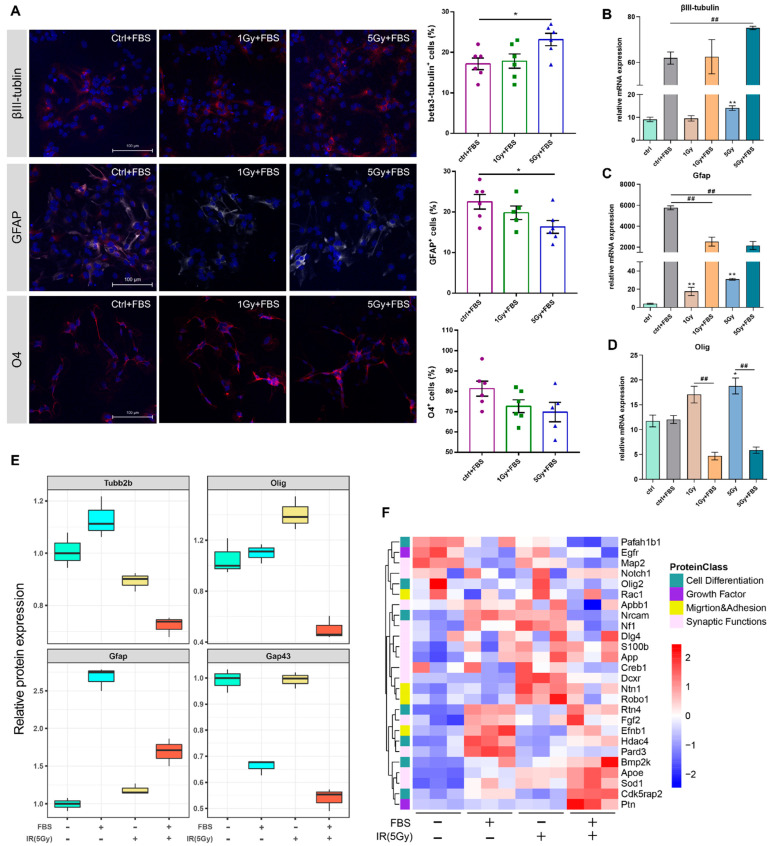
Assessment of differentiation in irradiated NSCs and visualization of neurogenesis-related proteins. (**A**) The confocal images depict representative immunostained cells for each phenotypic marker. Bar graphs depicting the expression percentages; data were represented as Mean ± SEM; *n* = 3; * *p* < 0.05 as compared with the indicated group. (**B**–**D**) Bar graphs representing the relative mRNA expressions of phenotypic marker genes. *n* = 3; Mean ± SEM. * *p* < 0.05, ** *p* < 0.01 as compared with the control group; ## *p* < 0.01 for comparisons between indicated groups. (**E**) Boxplot graph illustrating the relative expressions of cell-specific proteins selected from the MS/MS dataset. (**F**) Heatmap indicating the relative protein expressions among different treatment groups (red, upregulated; blue, downregulated), differently annotated color blocks represent different classes of the proteins.

Neural stem cell fate decisions are crucial for neurodevelopment and neurogenesis, which may contribute to cognitive processes, especially in irradiated brains. In order to address whether irradiation affects NSC differentiation at a protein level, NSCs were pre-treated with or without X-ray irradiation (1 Gy or 5 Gy), then allowed to differentiate in an FBS-containing DMEM medium. The mRNA expressions of cell type markers were significantly altered 24 h after DMEM + FBS medium replacement. The mRNAs of βIII-tubulin and GFAP, neuron and glial cell markers, were markedly upregulated in the FBS group, indicating that the NSCs were beginning to differentiate. When irradiation was involved, βIII-tubulin expression slightly increased, while GFAP expression diminished (Figure 4A–C). Conversely, Olig expression was not affected by FBS stimulation, and only 5 Gy irradiation upregulated its mRNA expression level. Following combination treatment with irradiation and FBS (1Gy+FBS and 5Gy+FBS groups), Olig2 expression decreased significantly (Figure 4D). Overall, during FBS-induced differentiation, irradiation interfered with the expression of cell-type marker genes by suppressing the expression of glial cells and oligodendrocyte-specific genes and promoting neural marker gene expression.

To further validate the expression pattern of these marker genes, the corresponding protein expression data were selected from our MS/MS spectra dataset. GFAP and Olig protein expressions were consistent with their mRNA expressions (Figure 4C–E). βIII-tubulin was not detected in MS/MS, but another neuron-specific protein was identified: tubb2b. In FBS-induced differentiation, irradiation significantly downregulated tubb2b expression (Figure 4E). Meanwhile, FBS-induced differentiation was conducted for 5 days, and the irradiation-induced NSC lineage commitment was evaluated with immunofluorescence. Similar to mRNA and protein results, more cells were beta3-tubulin^+^, and IR decreased gliagenesis and oligodendrogenesis (Figure 4F). Concurrently, the neurogenesis protein profile was identified by proteome analysis.

As illustrated in the heatmap, the neurogenesis-related proteins’ expressions among each group were clearly distinguished. Notably, ptn and cdk5rap2, which were demonstrated to be associated with Alzheimer’s disease, were upregulated in the IR+ differentiation group. Additionally, SOD1, the oxidative stress-related protein, promotes amyotrophic lateral sclerosis (ALS) [35]. HDAC4 was only upregulated in the differentiation group (DMEM+FBS) and was downregulated by irradiation. The inhibition of HDACs may impair neural stem cell activity [36]. Nrcam (neuronal cell adhesion molecule), a protein essential for neuron-neuron adhesion and which was also reported to be related to autism [37], was significantly downregulated in the IR + differentiation group. Pafah1b1, which was significantly downregulated in the IR + differentiation group, is a gene critical for brain development and is responsible for Lissencephaly [38]. Irradiation could hamper the proper differentiation of NSCs, and drive neurogenesis-related proteins to be expressed in a pattern of neurological diseases.

### 2.5. Construction of the Redox-Protein Profile in Irradiated NSCs Via Iodoacetyl-Labelled Mass Spectrometry

**Figure 5:**
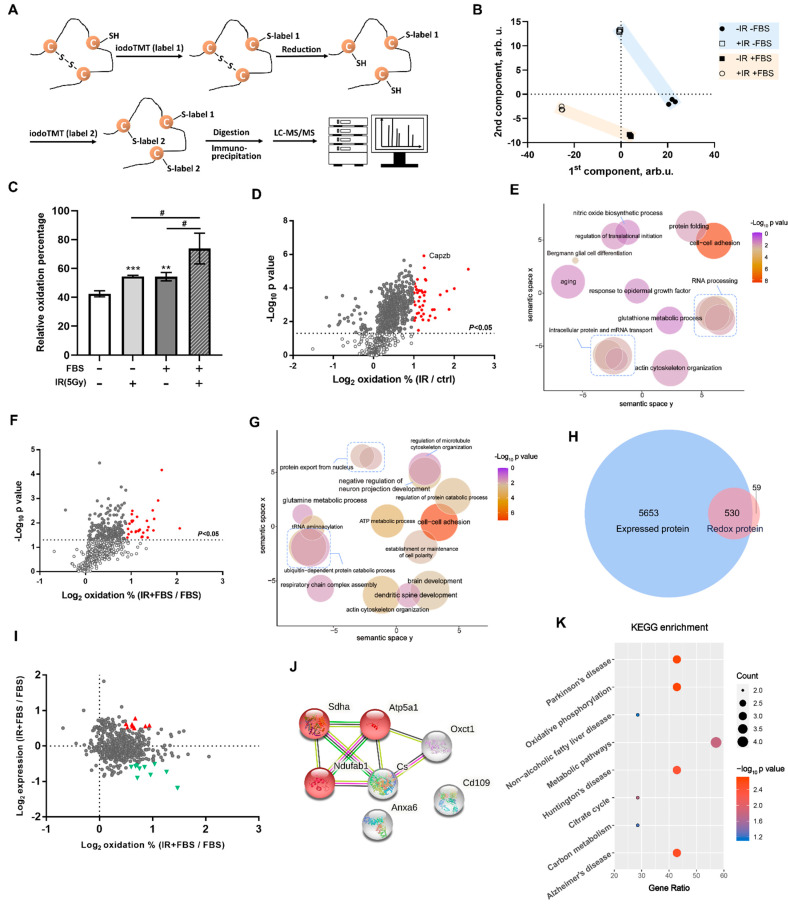
Redox protein detection and expression-oxidation conjoint analysis. (**A**) Schematic depiction of disulfide bond reduction workflow and free thiol-labeled oxidation proteomic screening. (**B**) Principal component analysis (PCA) of proteomic oxidation profile among groups, “–FBS–IR” represents the “ctrl” group, “–FBS+IR” denotes the “5Gy_ctrl” group, “+FBS–IR” represents the “FBS_ctrl” group, and “+FBS+IR” represents the “5Gy+FBS_ctrl” group. (**C**) Bar Graph showing the relative oxidation percentage of proteome-wide sulfhydryl groups among different treatment groups; data were represented as Mean ± SEM; *n* = 3; ** *p* < 0.01, *** *p* < 0.001 as compared with the “–IR–FBS” group, # *p* < 0.05 for comparisons between indicated groups. (**D**) Volcano plots exhibit the oxidation levels of detected proteins, the Y-axis represents the negative log10 of the *p* value, and the X-axis represents the log2 of the oxidation fold change between the IR and control groups. (**E**) The scatter plots illustrate the significantly enriched GO terms for oxidized proteins between the IR and control groups. (**F**) Volcano plots depict oxidation levels of detected proteins, the Y-axis represents the negative log10 of the *p* value, and the X-axis represents the log2 of the oxidation fold change between the IR+FBS and FBS groups. (**G**) Scatter plots demonstrating the significantly enriched GO terms for oxidized proteins between the R+FBS and FBS groups. (**H**) The Venn graph depicts the overlap of detected proteins between the expressed (TMT label) and redox datasets (iodoTMT label). (**I**) Scatter plot illustrating the co-detected proteins between the expressed and redox datasets; the Y-axis represents the log2 of proteins expression fold change between the IR+FBS and FBS groups, while the X-axis represents the log2 of the oxidation fold change between the IR+FBS and FBS groups; small red triangles represent proteins which are both highly expressed and heavily oxidized. (**J**) STRING analysis of selected proteins’ interactions. (**K**) Bubble diagram displaying the significantly enriched KEGG items; the bubble’s size represents the number of genes involved in the KEGG pathway, while the gradient colors represent the negative log10 of the *p* value.

In the previous section, the GO enrichment analysis revealed that the proteins specific for irradiated NSCs’ proliferation were tightly associated with oxidative stresses. Therefore, we next aimed to evaluate the extent of redox and identify the proteins with vital roles. As illustrated in the schematic diagram, the iodoTMTs were initially utilized to label all protein homogenates; thus, the basal level of free sulfhydryl groups among each treatment group could not be assessed (label 1). Subsequently, disulfide bonds were reduced, and the released sulfhydryl groups were classified as label 2. The intensities of label 1 and label 2 proteins were detected via MS/MS. The relative oxidation levels were obtained by calculating the label 2/ (label 1 + label 2) ratio and protein samples were collected 36 h after each treatment (Figure 5A). Data for co-expressed proteins were merged, and relative oxidation state proportions among different experimental groups were counted. Principal component analysis (PCA) indicated that the redox level of the same protein varied based on the different treatments (Figure 5B). Furthermore, the total label 2/(label1+label2) index in each group was calculated, and the relative oxidation percentage of the FBS+IR group was significantly higher than for other treatments (Figure 5C). 873 proteins were detected in both the control and IR group. Compared to ctrl, the log2 redox percentages of most irradiated proteins (766) were greater than zero (Figure 5D). This demonstrated that irradiation induced a more substantial increase in protein oxidation activation in NSCs. Significantly oxidized proteins were chosen for an analysis of their bio-functions; the oxidized proteins were enriched for aging, cell differentiation, cell adhesion, and RNA processing (Figure 5E). Importantly, evident oxidation also occurred in differentiating NSCs: 589 proteins were identified in the FBS and FBS + IR groups, and 92.5% of these proteins (545) were oxidized (Figure 5F). GO analysis indicated that significantly oxidized proteins could regulate neurons, brain development, cell adhesion and polarity, and cytoskeleton organization (Figure 5G). This strongly suggested that when irradiation disrupts the normal NSC differentiation process, these oxidized proteins play a deleterious function during this disruption. Subsequently, detectably expressed proteins and redox proteins in NSCs under IR+FBS treatment were compared; 589 redox proteins and 6183 expressed proteins were detected, respectively (Figure 5H). The two protein clusters shared 530 common proteins, and 59 were proteins exclusively detected by the redox method. GO enrichment of these 59 proteins did not yield significant evidence, only general information such as heterocyclic compound binding and ion binding function (Figure S1). Another notable concern was the association between upregulated and oxidized proteins after IR+FBS treatment. The 530 common proteins were analyzed using expression fold change (FC) and the percentage of oxidation (Figure 5I). At thresholds of |log2FC| > 0.5 and log2 oxidation % > 0.5, 8 upregulated and 10 downregulated oxidized proteins were identified. The biological processes and KEGG enrichment of the downregulated oxidated proteins focused predominantly on synapses, postsynaptic density, nitrogen compound metabolism, and ribosomes (Figure S2). Interestingly, the upregulated oxidized proteins possessed a strong association with the neurodegenerative pathways in KEGG, such as those involved in Parkinson’s disease, Huntington’s disease, and Alzheimer’s disease (Figure 5J,K). Overall, in post-irradiation exposure to NSC differentiation, certain highly expressed proteins were also considerably oxidized, indicating the activation of reverse pathways that may culminate in NSC degeneration.

## 3. Discussion

Ionizing radiation of the developing or adult brain is acknowledged as a potential cause of cognitive impairment and neurodegeneration, particularly when neural stem cells are affected [39,40,41,42]. Elucidating the mechanism underlying irradiation-mediated NSC injury would contribute to alleviating the side effects of radiotherapy and demystifying the induction of neural inflammation, brain development, and even neurodegeneration-associated mechanisms [43,44,45]. The substitution models employed in preclinical radiation research vary from cultured cells to small or large animals [46,47]; the majority of these models have been established according to the linear quadratic (LQ) model [48]. Currently, the emergence of 3D tissue models and organoids has been beneficial in understanding radiation-induced tissue response and in precision medicine [49,50].

With the advent of sequencing technology, neural stem cells have been investigated from a system-wide perspective, including transcriptomics, proteomics, and metabolomics, shedding new light on their complex regulatory mechanism [51,52,53,54]. Taking into consideration the properties and limitations of radiobiology models, as well as the complexity of the neural stem cell microenvironment in the brain, we sought to determine how NSCs respond to X-ray irradiation stress in the absence of cellular interactions. Therefore, in the present study, we designed a neural stem cell in vitro radiation model and integrated expression and redox proteomic techniques to analyze global protein expression in differentiated neural stem cells following X-ray irradiation. The proteomic expression profile demonstrated that irradiation impaired NSC proliferation, the cell cycle, and differentiation; in particular, the oxidation of those upregulated proteins posed an extremely high risk of neurodegeneration.

Proliferating neural stem cells or progenitor cells are tremendously sensitive to ionizing radiation-induced DNA damage and apoptosis [55,56]. This phenomenon was also reflected in our BrdU assay. When DNA damage is induced, the replication checkpoint initiates the DNA repair response and delays the cell cycle progress. In neural stem cells, the cell cycle is also associated with cell differentiation: prolonged G1 and upregulated p57 enable cells to respond to signals rapidly and differentiate properly [56,57,58,59]. The manipulation of the G1 phase by CDKs could regulate the NSCs’ fate, proliferation, or differentiation [60,61,62]. After irradiation, the G1 phase was shortened in differentiating NSCs, suggesting that irradiation disrupted the conditions for normal NSC differentiation. Nestin, an intermediate filament protein, is universally considered a marker of neural stem/progenitor cells [63]. Upregulated nestin expression was detected in stem/progenitor cells during the early development stage in which cells are engaged in active proliferation. Once these cells ceased dividing and initiated differentiation, nestin expression became downregulated [64]. Nestin expression is representative of NSCs’ pluripotential. It has been reported that irradiation significantly reduced the nestin-positive cells in the mouse brain’s dentate gyrus [65]. When co-cultured with irradiated vascular endothelial cells, nestin-positive NSCs exhibited a marked decline [66]. In this study, nestin mRNA expression was similarly downregulated when NSCs were subjected to X-ray irradiation, indicating a deleterious effect of irradiation on NSCs’ stemness. Under pathological conditions, nestin should be re-expressed for the repair process to be initiated [67]. However, it is difficult to determine whether those nestin-deficient NSCs are capable of completing the repair task in irradiation-induced brain injury.

The predominant cytotoxic effects of irradiation are DNA damage and cell cycle arrest [68,69]; another adverse effect of irradiation that could cause cognitive impairment is reduced neurogenesis. Irradiation induces apoptosis in dividing cells, reduces the pool of mitotic NSCs, hampers the generation of new neurons [3], affects the microenvironment of the targeted brain tissue site, and alters the NSC niche [70,71]. The expression of neurogenesis-related proteins in our proteomic datasets also reflected the detrimental effects of irradiation. Cyclin-dependent kinase 5 regulatory subunit-associated protein2 (CDK5RAP2) has been implicated in the proliferation of neuronal progenitors in the developing neocortex [72] and was also shown to cause Seckel syndrome [73]. Justin Miron et al. reported that CDK5RAP2 was prevalent in the hippocampus of brains that develop Alzheimer’s disease (AD). Notably, we also detected increased CDK5RAP2 expression in irradiated NSCs. Similar characteristics seem to occur for other neurological disease-related genes. Appb1, which interacts with amyloid precursor protein in Alzheimer’s disease, was downregulated in irradiated NSCs. Appb1 deletion was discovered to increase the risk of AD [74]. Likewise, Appb1 knockout in mice resulted in impaired learning and memory [75]. SOD1, a superoxide scavenger, is frequently upregulated during redox reactions [76]. We found that SOD1 was upregulated in irradiated NSCs, and chiefly attributed this to the IR-induced ROS. SOD1 was also upregulated in amyotrophic lateral sclerosis (ALS) patients [77], which indicates potential connections between irradiation and neurodegenerative disorders. Nrcam, a cell adhesion molecule, has been associated with autism spectrum disorders (ASD) [78]. Nrcam-knockout mice demonstrated autism-related behaviors, such as impaired sociability, cognitive rigidity, and repetitive behavior [79]. In the present study, Nrcam expression was also decreased in irradiated NSCs. Numerous neurogenesis-associated proteins altered by IR could not all be listed here. Nevertheless, IR’s impact on NSCs is considerably more complex than appreciated, especially the potential risk for neurodegeneration.

Beta tubulin III, also known as Tuj-1, a class III member of the beta tubulin protein family, is regarded as a neuron-specific marker to detect progenitor cell differentiation. Consistent with Hyeon Soo Eom et al.’s study [80], we observed upregulated Tuj-1 in irradiated NSCs. MAP2, another neuron marker, was upregulated in our MS/MS detection; however, Anggraeini Puspitasari et al. demonstrated that MAP2 expression was upregulated during the early stage of irradiation (4 days) and progressively diminished in the subsequent 20 days [81]. Recent research suggested that the two markers belong to two distinct types of neurons: Tuj1 are from pan-neurons, and MAP2 are from mature neurons [82]. The inconsistency between results for Tuj-1 and MAP2 expressions indicated that during NSCs differentiation, IR’s effects on neurons might vary depending on cell types; nonetheless, the specific mechanisms warrant further investigation.

Reactive oxygen species (ROS), a group of aerobic respiration metabolic byproducts, are responsible for cellular redox homeostasis. During exposure to ionizing radiation, abundant quantities of ROS and reactive nitrogen species (RNS) are generated by extracellular water radiolysis and mitochondrial membrane destruction [83,84]. ROS and RNS are the principal sources of oxidative damage to normal tissues. Concurrently, excessive ROS or RNS causes the oxidation of lipids, DNA, and proteins [85,86,87]. The oxidation of protein cysteine by ROS or RNS has been recognized as a prominent class of protein posttranslational modifications, which are heavily associated with aging and multiple diseases [88,89,90]. Two kinds of protein oxidative modifications exist irreversible oxidation and reversible oxidation. Irreversible oxidation results in protein dysfunction. In comparison, reversible oxidation, primarily of cysteine residues, could regulate the activity, the redox balance, and signaling cascades [91,92]. In the present study, we utilized cysteine-reactive tandem mass tags (iodo TMT) to detect reversible oxidation. The LC-MS/MS data could provide a proteome-wide protein oxidation profile beneficial for the analysis of the adverse effects of IR-induced oxidative stress.

Ionizing radiation significantly elevated the protein oxidation level in differentiating NSCs. The proteins with a dual increase in expression and oxidation levels, especially Sdha, Atp5a1, and Ndufab1, have been documented in studies of neurodegenerative diseases [93,94,95,96,97]. Nevertheless, the oxidation of these disease-marker proteins received scant attention. IR-induced proteome-wide protein oxidation could be associated with an increased risk of neurodegeneration, whereas limiting the oxidation of certain risk proteins would provide an auxiliary strategy for alleviating radiotherapy-induced brain injury.

In recent years, the majority of patients worldwide have turned toward photon therapy, and the utilization of charged particle therapies, including proton and carbon ion therapy, has substantially expanded [98]. Particle therapy treatment could substantially diminish the exposure of healthy tissue to radiation and long-term side effects [99,100], particularly among pediatric patients, in whom exposure of healthy organs to radiation doses can induce long-term detrimental effects [99]. We also have been conducting a collaborative Boron neutron capture therapy (BNCT) research project with the institute of high energy physics of the Chinese Academic of Sciences (CAS). Referring to economic considerations and indications such as meningiomas, ionizing radiation still has clinical utility. Investigations of radiation-induced injury could enable a deeper understanding of our coping mechanism when subjected to stressful radioactive rays and the progression of senescence. It is anticipated that the survival rates of cancer patients will continuously improve due to the constant evolution of modern radiotherapy.

## 4. Materials and Methods

### 4.1. Cells and X-ray Irradiation

GFP-transfected C57BL/6 mouse neural stem cells (NSCs), derived from 12.5 dpc embryos, were purchased from Cyagen Biosciences (MUBNF-01101, Guangzhou, China). The NSCs were maintained in a humidified incubator with 5% CO_2_ at 37 °C in Cyagen recommended medium (OriCellTM Neural Stem Cell Growth Medium, MUCMX-90011). The medium was changed every 2 days. Oricell Neural stem cell growth medium was replaced by 10% fetal bovine serum (FBS)/DMEM-F12K (Gibco) for differentiation. For X-ray irradiation (IR) treatment, the cells were irradiated at 1 Gy or 5 Gy with an Xstrahl X-ray system, Model CIX2 (Xstrahl, Walsall, West Midlands, UK). The follow-up procedures are described in subsequent sections.

### 4.2. qRT-PCR Analysis

The experiment was conducted for six groups, namely: ctrl, differentiation group (NSCs treated with FBS), irradiation group (cells exposed to X-ray,1 Gy or 5 Gy), differentiation after IR group (after 1 Gy or 5 Gy irradiation, the culture medium was immediately changed to FBS/DMEM-F12K). Total RNA extraction was performed at 24 h post-X-ray irradiation or cell differentiation using TRIzol Plus RNA kit (Invitrogen, Carlsbad, CA, USA). cDNA was prepared using the iScript cDNA synthesis kit (Bio-Rad, Hercules, CA, USA). The RT-PCR reaction was performed using Universal SYBR Green Supermix (Bio-Rad, Hercules, CA, USA) according to the manufacturer’s instructions. Information on the primers is listed in Supplementary Table S1. The statistical analysis was performed using GraphPad Prism 8.0 Software. The results were presented as the Mean ± standard error of the mean. Student’s t-test was used to compare values between the two groups. Differences were considered statistically significant when *p* values were <0.05.

### 4.3. Cell Cycle Analysis

The experimental groups and study design were consistent with the statements mentioned above. 24 h after each specific treatment, cells were collected via trypsinization. Furthermore, supernatants and PBS used during wash steps were kept ensuring the collection of both adherent and detached cells. After collection, the cells were fixed in ice-cold 70% ethanol at 4 °C overnight. Subsequently, the cells were stained with PI solution (50 μL PI and 50 μL RNase A in 10 mL PBS) for 30 min at room temperature before measurement. The data were obtained using a flow cytometer (Beckman Coulter, Brea, CA, USA) and analyzed using the ModFitLT software (Version 5.0; Verity Software House, Topsham, ME, USA).

### 4.4. BrdU Assay

NSCs were seeded in 96-well plates and subjected to specific stimulation (mentioned above). 24 h after treatment, the NSCs’ proliferation in each well was evaluated using a Cell Proliferation ELISA BrdU Kit (Roche, Mannheim, Germany) according to the manufacturer’s protocol. The absorbance, which represents BrdU incorporation during DNA synthesis, was measured at 450 nm using a microplate spectrophotometer (Thermo, Swedesboro, NJ, USA)

### 4.5. Immunofluorescence Staining

Neurospheres were trypsin-digested into a single-cell suspension and cultured on 0.01% poly-L-lysine (Sigma-Aldrich, St. Louis, MO, USA) pre-coated coverslips in a 24-well plate. The cells were induced to differentiate following 0 Gy, 1 Gy, or 5 Gy irradiation. After 5 days of differentiation, the cells were fixed with 4% Paraformaldehyde (PFA), followed by PBS washing thrice and blocking for 1 h with 0.5% bovine serum albumin (BSA) and 0.1%Triton X-100. The blocking solution was also used for antibody dilution: Rabbit anti-GFAP (1:1000, Abcam, Cambridge Biomedical Campus, Cambridge, UK), Mouse anti-O4 (1:1000, R&D systems, Minneapolis, MN, USA), and Mouse anti-beta 3 tubulin (1:1000, Sigma), and the primary antibodies were incubated at 4 °C overnight. After several washes with TBS, the corresponding secondary antibodies were added for 2 h at room temperature. The utilized secondary antibodies are as follows: Donkey anti-mouse IgM Alexa 555 and Donkey anti-rabbit IgM Alexa 633 (Thermo, Waltham, MA, USA). The cell climbing slices were mounted on glass slides with an antifade reagent mounting medium (BOSTER Biological Tech, Wuhan, China). All the stained fluorescent markers were captured using an LSM 700 laser scanning confocal microscope (Axio-observer Z1; Carl Zeiss, Oberkochen, Germany) and analyzed using the software ZEN lite (Zeiss, https://www.zeiss.com/microscopy/en/products/software/zeiss-zen-lite.html/, accessed on 18 March 2020)

### 4.6. Protein and LC MS/MS and TMT Label

Protein sample preparation. 36 h after corresponding treatments, all NSC samples were lysed in RIPA buffer with PMSF (Abcam), then centrifuged at 12,000× *g* for 10 min at 4 °C; the supernatants containing total proteins were collected. The protein concentration per sample was determined using Pierce BCA Protein Assay Kit (Thermo scientific, Rockford, IL, USA) according to the manufacturer’s protocol. Aliquots of 50 μg proteins were used for proteomics analysis. Proteins’ disulfide bonds were reduced with 10 mM Dithiothreitol for 45 min at 55 °C, then alkylated with 25 mM iodoacetic acid for 30 min in the dark, followed by overnight acetone precipitation. The obtained precipitants were dissolved in EPPS (Thermo Fisher Scientific, Rockford, IL, USA) and re-dissociated with Trypsin overnight at 37 °C. Peptide and the sulfhydryls of cysteine-containing peptides labeling were performed using TMT10-plex and iodoTMT Mass Tag Labelling Kit (Thermo Fisher Scientific, Rockford, IL, USA) following the manufacturer’s protocol. The labeled samples were acidified with trifluoroacetic acid, followed by a desalination procedure using a C18 Sep-pak column, and then vacuum dried.

LC-MS/MS analysis. The peptide samples were dissolved in 0.1% formic acid, then preconcentrated and desalted using PepMap C18 nanotrap column (Thermo Fisher Scientific, Rockford, IL, USA) A reversed-phase analytical column (EASY-Spray C18, Thermo Fisher Scientific, Rockford, IL, USA) was utilized for peptide separation in a binary solvent system. Gradient conditions were: 4–26% solvent B for 120 min and 26–95% B for 10 min. The peptides were analyzed using a data-dependent acquisition method at a resolution of 120,000, a scan range of 375–1500 *m*/*z*, and at a resolution of 60,000 with a target value of 2 × 10^5^ ions and a maximum injection time of 120 ms. The fixed first *m*/*z* was 100, and the isolation window was 1.2 *m*/*z* units. The raw data files were processed using the Andromeda search engine in MaxQuant 1.5.6.5 software (https://www.maxquant.org/, accessed on 11 November 2019)

### 4.7. Bioinformatic Analysis

All statistics of protein expression data were computed using Excel software (Microsoft Excel, version 2013), and the differentially expressed (DE) proteins were screened via the t-test (p < 0.05) and Log2 fold change (Log2fold change>|0.5|); related expression volcano plots were generated using GraphPad Prism V 7.0. The clustered heatmap profile of protein expression among each group was conducted using the “pheatmap” package (version 1.0.12, https://cran.rstudio.com/web/packages/pheatmap/index.html/, accessed on 15 February 2022) in R.

The principal component analysis of protein expression patterns among groups was performed using the “FactoMineR” package (version 2.4, https://cran.r-project.org/web/packages/FactoMineR/index.html/, accessed on 15 February 2022) in R, and the output data were plotted using GraphPad Prism. For correlation analysis, the normalized protein expression values of particular experimental groups were transformed on a Log2 scale, then analyzed and visualized with GraphPad Prism.

The gene ontology (GO) or KEGG enrichment of DE proteins among experimental groups was performed using the online database DAVID (https://david.ncifcrf.gov/, accessed on 13 February 2022). Protein interactions were analyzed using the STRING database (http://string-db.org/, accessed on 13 February 2022). The obtained GO data (p-value <0.05) were visualized using the REVIGO web server (https://revigo.irb.hr/, accessed on 13 February 2022). The KEGG enrichment scattered plots were generated utilizing the R packages ggplot2 (version 3.3.5, https://www.rdocumentation.org/packages/ggplot2/versions/3.3.5/, accessed on 10 April 2022).

## 5. Conclusions

We utilized mouse neural stem cells to establish an X-radiation injury cell model and introduced FBS to simulate the differentiation process. Mass spectrometry protein profiling and redox proteomic techniques were applied to analyze global protein expression in differentiated neural stem cells upon X-ray irradiation. LC-MS/MS permitted the detection of a series of significantly expressed proteins related to alterations of the cell cycle, impaired proliferation, and differentiation in NSCs. These results evidenced the deleterious effects of irradiation on neural stem cells at a protein level. Furthermore, we first employed iodoTMT labeling techniques to obtain a redox protein profiling of differentiating NSCs under irradiation stress. The joint analysis of expressed and redox protein profiles have identified highly upregulated and oxidized proteins associated with neurodegenerative disease. From a redox perspective, irradiation could impede the normal processes involved in NSC differentiation, thereby resulting in degenerative differentiation.

## 6. Patents

This section is not mandatory but may be added if there are patents resulting from the work reported in this manuscript.

## Data Availability

Not applicable.

## Supplementary Materials

The following supporting information can be downloaded at: https://www.mdpi.com/article/10.3390/biom12121759/s1, Figure S1: STRING analysis of selected protein interactions, Figure S2: STRING analysis of selected protein interactions, Table S1: Primer information.
