# Supplementary material for: Radiotherapy Side Effects: Comprehensive Proteomic Study Unraveled Neural Stem Cell Degenerative Differentiation upon Ionizing Radiation
Source: Biomolecules. 2022 Nov 26;12(12):1759. doi: 10.3390/biom12121759 (PMC9775306; doi:10.3390/biom12121759)
Supplement: Supplementary file 1 [file biomolecules-12-01759-s001.zip › Table S1.pdf]

Table S1. Primer information

Gene Name	Sequence (5'-3')	Product length (bp)
Nestin	F: CTCGAGCAGGAAGTGGTAGG	140
	R: GCCTCTTTTGGTTCCTTTCC	
Sox2	F: GAACGCCTTCATGGTATGGT	125
	R: TCTCGGTCTCGGACAAAAGT	
Neurog1	F: CACCACTCTCTGACCCCAGT	109
	R: GTCGTGTGGAGCAGGTCTTT	
Ki67	F: ATCCAGATGATGGAGCCAAG	139
	R: ATTTCTGCAGCTGGTTTGCT	
p57	F: GGAGCAGGACGAGAATCAAG	145
	R: GTTCTCCTGCGCAGTTCTCT	
p21	F:ACGGTGGAACTTTGACTTCGTC	214
	R:CAGAGTGCAAGACAGCGACAAG	
p27	F: GCGGTGCCTTTAATTGGGTCT	230
	R: GGCTTCTTGGGCGTCTGCT	
βIII-tubulin	Qiagen, QT01227128	85
Olig2	Qiagen, QT01041089	73
Gfap	Qiagen, QT00101143	127
GAPDH	Qiagen, QT01658692	144
