# Supplementary figures and images for: Radiotherapy Side Effects: Comprehensive Proteomic Study Unraveled Neural Stem Cell Degenerative Differentiation upon Ionizing Radiation
Source: Biomolecules. 2022 Nov 26;12(12):1759. doi: 10.3390/biom12121759 (PMC9775306; doi:10.3390/biom12121759)

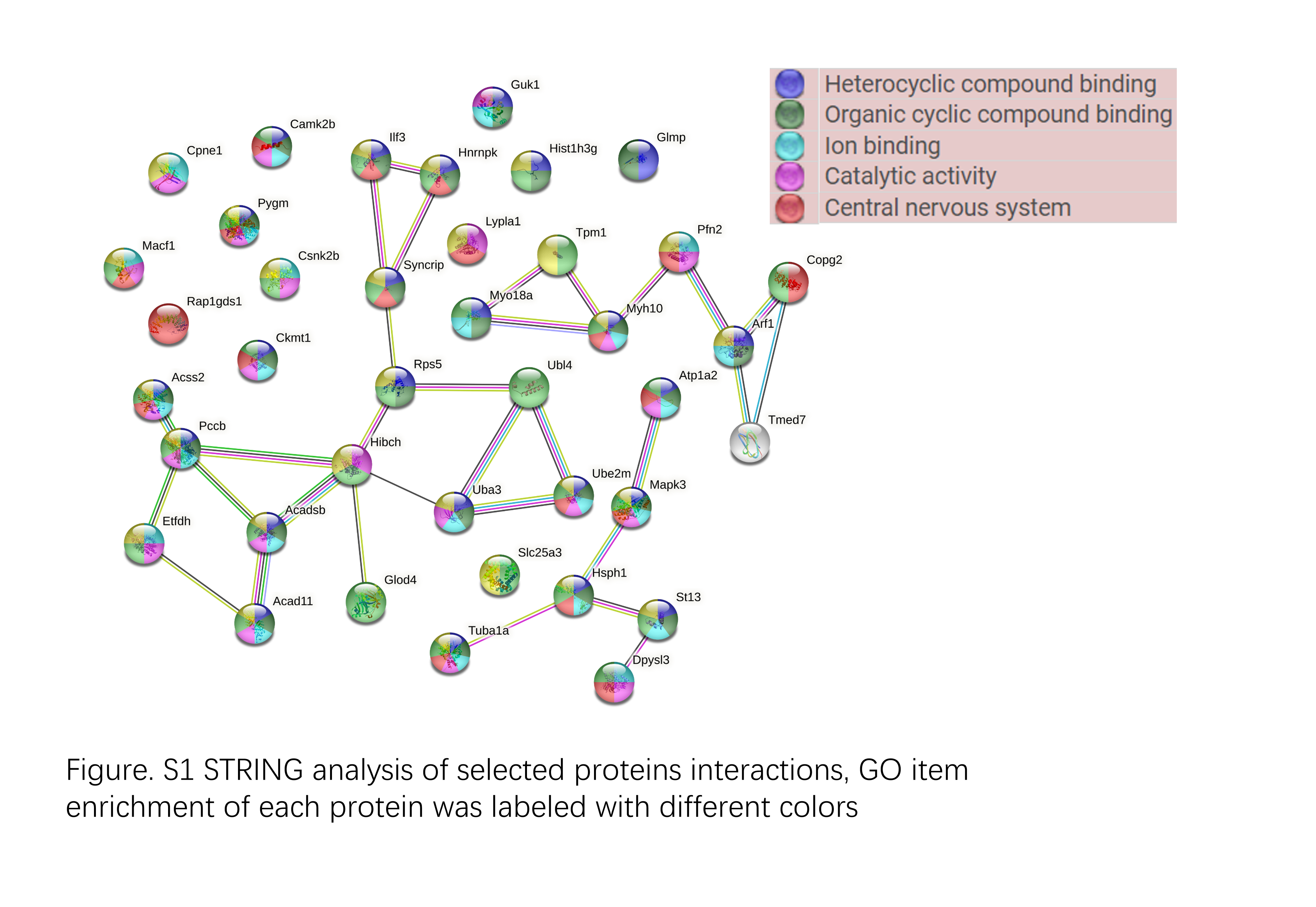

Supplement: Supplementary file 1 [file biomolecules-12-01759-s001.zip › Figure S1.png]

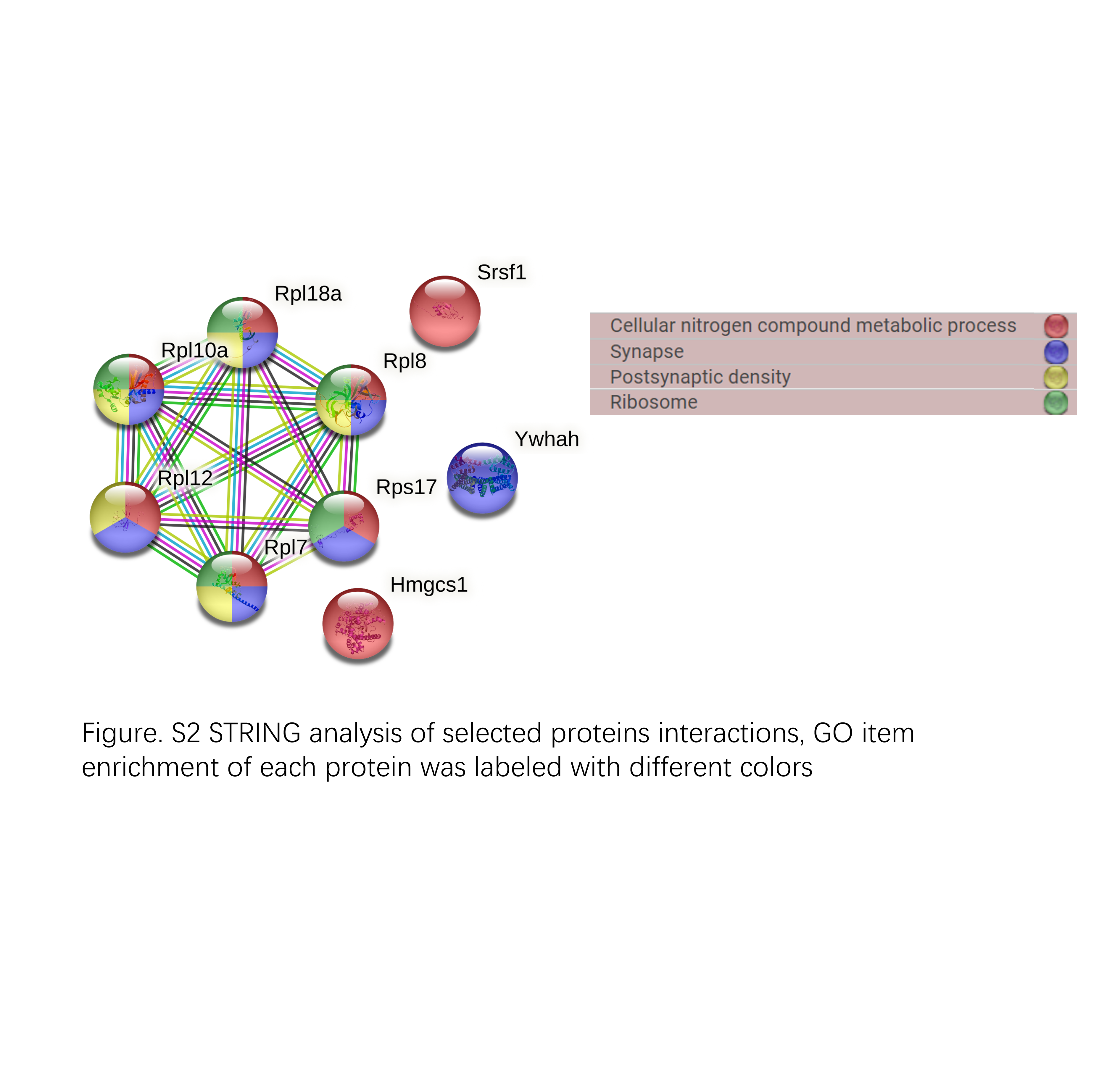

Supplement: Supplementary file 1 [file biomolecules-12-01759-s001.zip › Figure S2.png]
